# Leptomeningeal disease in histone-mutant gliomas

**DOI:** 10.1093/noajnl/vdad068

**Published:** 2023-05-29

**Authors:** Maria Diaz, Satshil Rana, Carlos Eduardo Silva Correia, Anne S Reiner, Andrew L Lin, Alexandra M Miller, Maya S Graham, Sofia Chudsky, Tejus A Bale, Marc Rosenblum, Matthias A Karajannis, Elena Pentsova

**Affiliations:** Department of Neurology, Memorial Sloan Kettering Cancer Center, New York, NY, USA; Department of Pathology, Memorial Sloan Kettering Cancer Center, New York, NY, USA; Department of Neurology, Memorial Sloan Kettering Cancer Center, New York, NY, USA; Department of Epidemiology and Biostatistics, Memorial Sloan Kettering Cancer Center, New York, NY, USA; Department of Neurology, Memorial Sloan Kettering Cancer Center, New York, NY, USA; Department of Neurology, Memorial Sloan Kettering Cancer Center, New York, NY, USA; Department of Neurology, Memorial Sloan Kettering Cancer Center, New York, NY, USA; Office of Professional Development, Memorial Sloan Kettering Cancer Center, New York, NY, USA; Hunter College, New York, NY, USA; Department of Pathology, Memorial Sloan Kettering Cancer Center, New York, NY, USA; Department of Pathology, Memorial Sloan Kettering Cancer Center, New York, NY, USA; Department of Pediatrics, Memorial Sloan Kettering Cancer Center, New York, NY, USA; Department of Neurology, Memorial Sloan Kettering Cancer Center, New York, NY, USA

**Keywords:** cerebrospinal fluid, circulating tumor DNA, diffuse hemispheric glioma, diffuse midline histone mutations, glioma, leptomeningeal disease

## Abstract

**Background:**

The 2016 WHO classification described a subtype of midline gliomas harboring histone 3 (H3) K27M alterations, and the 2021 edition added a new subtype of hemispheric diffuse gliomas with H3 G34R/V mutations. The incidence and clinical behavior of leptomeningeal disease (LMD) in these patients is not well defined.

**Methods:**

Retrospective study of patients with H3-altered gliomas diagnosed from 01/2012 to 08/2021; histone mutations were identified through next-generation sequencing (NGS) of tumor biopsy and/or cerebrospinal fluid (CSF).

**Results:**

We identified 42 patients harboring H3 mutations (K27M mutations in 33 patients, G34R/V in 8, and both in one). Median age was 21 (4–70); 27 were male. LMD was diagnosed in 21/42 (50%) patients, corresponding to a 3-year cumulative incidence of 44.7% (95% confidence interval (CI): 26.1%–63.4%) for the K27-mutant group and a 1-year cumulative incidence of 37.5% in the G34-mutant group (95% CI: 0.01%–74.4%; no events after 1 year). Median time from tumor diagnosis to LMD was 12.9 months for H3-K27 patients and 5.6 months for H3-G34 patients. H3 mutation was detected in CSF in all patients with LMD who had NGS (8 H3-K27-mutant patients). In the H3-K27-mutant group, modeled risk of death was increased in patients who developed LMD (hazard ratio: 7.37, 95% CI: 2.98–18.23, *P* < .0001).

**Conclusions:**

In our cohort, 50% of patients developed LMD. Although further studies are needed, CSF ctDNA characterization may aid in identifying molecular tumor profiles in glioma patients with LMD, and neuroaxis imaging and CSF NGS should be considered for early LMD detection.

**Key Points**

- We conduct full clinical characterization in patients with non-H3K27-altered DMG.
- We show a higher risk of leptomeningeal dissemination in H3G34-altered DMG.
- We perform molecular characterization in tissue and CSF ctDNA in these DMG patients.

**Importance of the Study**

The risk of leptomeningeal dissemination (LMD) in primary brain tumors has been mostly studied in diffuse intrinsic pontine gliomas (DIPG), which only represent a subset of H3 K27-altered diffuse midline gliomas (DMG). Here we provide the full clinical characterization of a retrospective case series of patients with DMG harboring H3 K27 or H3 G34 alterations (or both), including survival, LMD, and next-generation sequencing of tumor biopsies and cerebrospinal fluid (CSF) ctDNA. Our findings demonstrate the unique characteristics of each molecular subgroup and suggest that H3 K27-altered DMG and H3 G34-altered hemispheric gliomas may be at higher risk of developing LMD. We also assess the value of CSF next generation sequencing in these patients. Overall, our data underscore the importance of conducting prospective studies considering these molecular entities as separate subgroups and point out to novel prognostic biomarkers for higher risk of primary brain tumor dissemination through the neuroaxis.

The World Health Organization (WHO) Classification of Tumors of the Central Nervous System (CNS) introduced diffuse midline glioma (DMG), H3 K27M-mutant as a new entity in 2016,^1^ and further refined the nomenclature to H3 K27-altered in the 2021 classification to reflect specific molecular mechanisms of pathogenesis.^2^ These aggressive tumors are defined by presence of genetic alterations in one of the genes that encode histone H3 (namely *H3F3A* and *HIST1H3B*), most commonly resulting in the substitution of lysine 27 to methionine (K27M). They involve midline cerebral structures and tend to affect children, adolescents, and young adults, although cases in older adults have also been described.^3^ The most recent WHO 2021 introduced a separate type of high-grade glioma, H3 G34-mutant diffuse hemispheric gliomas (DHG), which are defined by a different point mutation in codon 34 of histone H3, which causes the substitution of glycine to valine, G34V, or glycine to arginine, G34R. These tumors occur in the cerebral hemispheres, typically affect adolescent and young adult patients, and are also considered an aggressive tumor type; both H3 K27-altered and H3 G34-mutant diffuse gliomas are classified as WHO grade 4 regardless of histological characteristics.^4^

Through case reports and autopsy series, it has been shown that patients with DMG may be at an increased risk of tumor dissemination through the neuroaxis, with an incidence of leptomeningeal disease (LMD) of up to 40% in autopsy reports and over 50% in prospective clinical studies.^5,6^ However, most of the evidence supporting these observations is based on studies from patients with diffuse intrinsic pontine gliomas (DIPG), which only represent a subset of H3 K27-altered DMG patients^3^; therefore, the frequency and characteristics of LMD in non-DIPG H3 K27-altered DMG have not been characterized in depth. Moreover, the prevalence and patterns of leptomeningeal dissemination in patients with H3 G34-mutant diffuse hemispheric gliomas have not been described. The purpose of our present study was to investigate the occurrence of LMD in a retrospective cohort of glioma patients meeting the criteria for H3 K27-altered DMG or H3 G34-mutant DHG, and to describe the clinical characteristics and survival of the patients who developed LMD.

## Methods

### Patient Selection

This retrospective study was approved by the Memorial Sloan Kettering Cancer Center (MSK) Institutional Review Board. Informed consent was institutionally waived due to the retrospective nature of the study, which entails minimal risk to participating patients. Patients with H3 K27 or H3 G34 mutations were identified using the MSK DARWIN database, a research database linked to the electronic medical record, through next-generation sequencing (NGS) data resulting from either tumor tissue or cerebrospinal fluid (CSF) between January 2012 and August 2021. Charts were manually reviewed to select patients with a glioma diagnosis meeting the 2021 WHO classification characteristics of diffuse midline glioma, H3 K27-altered; or diffuse hemispheric glioma, H3 G34-mutant.^2^ A second MSK DARWIN database search was conducted for patients with a pathological diagnosis of glioma and positive H3 K27M on immunohistochemistry. For all selected patients, information regarding demographic characteristics, date of diagnosis, development of LMD, magnetic resonance imaging (MRI) and CSF results, treatments received, follow-up, and survival were collected. LMD diagnosis was based on brain and/or spine MRI, results of CSF cytology, or a combination of both, as per the RANO-LM criteria^7^; date of LMD diagnosis was determined as the date of either the first MRI that showed imaging signs of LM or the date of a positive/suspicious CSF cytology, whichever came first.

### Next-Generation Sequencing in Tumor Tissue and CSF

Patients underwent lumbar punctures, if indicated as part of their standard clinical care at MSK, for standard testing including CSF cytology. DNA was extracted from formalin-fixed, paraffin-embedded tumor tissue, and from CSF and underwent next-generation sequencing (NGS) through the Memorial Sloan Kettering-Integrated Molecular Profiling of Actionable Cancer Targets (MSK-IMPACT) assay.^8^ In the patients where CSF sequencing using MSK-IMPACT failed to demonstrate an *H3* mutation using the clinically validated thresholds, a secondary targeted mutation analysis was performed, as previously described in more detail in prior studies.^9^ The results of NGS in CSF of several patients in this study have been previously published in more detail in two other manuscripts by our group.^9,10^

### Statistical Analysis

Descriptive statistics (mean, median, and range) were used to characterize the cohort. Overall survival was estimated using Kaplan–Meier methodology and follow-up was defined from time of tumor diagnosis until death (event) or last follow-up (censored). Cumulative incidence of LMD was estimated in the competing risks setting with death as a competing event. Because development of leptomeningeal metastases (LM) is not known at baseline, the timing of LM must also be incorporated into the analysis. Therefore, univariable Cox proportional hazards modeling was performed with LM as a time-dependent variable. To visually depict the effect of LM on survival seen in the K27-mutated group, a complementary landmark analysis was performed using the median time to LM as the landmarked time. LM status prior to this time contributed to this figure and patients who had died or were lost to follow-up prior to this time were excluded from the figure. All tests were 2-sided with a level of statistical significance set at <.05. All analyses were performed in SAS version 9.4 (The SAS Institute, Cary, NC) and R v4.2.2 (The R Foundation for Statistical Computing).

## Results

A total of 42 eligible patients that had been subject to NGS (38 in tumor tissue and 4 in CSF) were included. There were 33 patients with H3 K27 mutations, 8 patients with H3 G34 mutations, and 1 patient with both mutations; given that the clinical characteristics of this patient were consistent with a diagnosis of diffuse midline glioma, H3 K27-altered (with a tumor located in thalamic location), this patient was grouped and analyzed with the H3 K27-mutant group.

**Figure 1.**
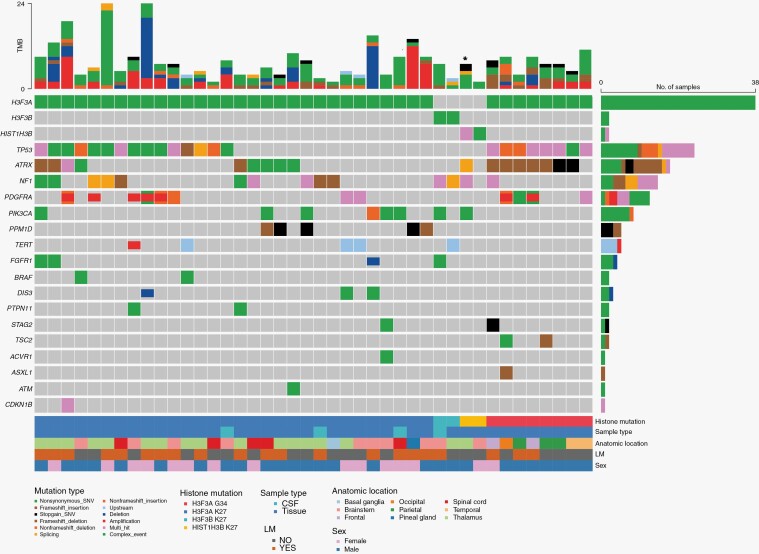
Oncoplot detailing next-generation sequencing results and clinicopathological characteristics of all 42 patients, organized by histone mutation. Copy number variations are represented by coloring of the middle portion of the square (deletions in red, amplifications in blue). For patients with both CSF and tissue NGS available, tissue results are included here; for 4 patients only CSF NGS was available. *Denotes patient with both K27 and G34 mutations.

Figure 1 summarizes NGS results along with clinicopathological information for the entire group of 42 patients. Whereas most patients in both groups had an *H3F3A* mutation, there were 2 patients with a *HIST1H3B* mutation, as well as 2 patients with an atypical mutation in *H3F3B* (K27I) in the *H3 K27* group, one of which was diagnosed through CSF NGS. The clinical picture of the patients with an *H3F3B* mutation fits the characteristics of a diffuse midline glioma: both were young patients diagnosed with expansile masses in midline locations (1 thalamic and 1 medullary) with histological characteristics of high-grade gliomas on tissue analysis. The second most commonly altered gene was *TP53*, with alterations found in 14/34 (41%) of H3 K27 and 8/8 (100%) of H3 G34 patients, followed by *ATRX* (10/34 [29%] and 7/8 [88%]), *NF1* (12/34 [35%] and 1/8 [13%]), *PDGFRA* (8/34 [18%] and 4/8 [24%]), *FGFR1* (4/34 [11%]) and BRAF (2/34 [5%]).

**Figure 2.**
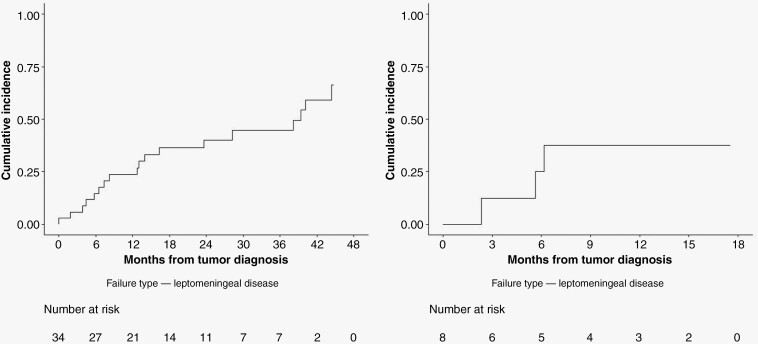
Cumulative incidence of leptomeningeal disease for H3 K27 patients (left) and H3 G34 patients (right).

Table 1 summarizes the clinical and pathological characteristics of the entire cohort. Median age was 21 years for both groups, and most patients were male (20/34 [59%] in the H3 K27 group and 7/8 [87.5%] in the H3 G34 group). All patients received treatment with at least radiation therapy, and most patients (41/42) received at least 1 line of chemotherapy (Supplementary Table 1). After a median follow-up of 19.3 months in the H3 K27 group, the 3-year cumulative incidence of LMD was 44.7% (95% CI: 26.1%–63.4%); following a median 15.1-month follow-up in the H3 G34 group, the 1-year cumulative incidence of LMD was 37.5% (95% CI: 0.01%–74.4%), respectively (Figure 2). We did not observe other LMD events after 1 year follow-up in the H3 G34 group. For comparison, the 1-year cumulative incidence of LMD for the K27 group was 23.7% (95% CI: 9.1%–38.2%).

**Table 1.** Clinical and pathological characteristics of all patients.

	H3 K27-altered diffuse midline glioma (*N* = 34)	H3 G34-mutant diffuse hemispheric glioma (*N* = 8)
Sex Female Male	14 (41.2%) 20 (58.8%)	1 (12.5%) 7 (87.5%)
Age in years, median (range)	21 (4–70)	21 (9-32)
Tumor location	Thalamus—17 (50%) Brainstem—10 (29.4%) Spine—5 (14.7%) Other*—2 (5.9%)	Frontal—2 (25%) Temporal—2 (25%) Parietal—3 (37.5%) Occipital—1 (12.5%)
Histone mutation	*H3F3A*—30 (88.2%) *H3F3B*—2 (5.9%) *HIST1H3B*—2 (5.9%)	*H3F3A*—8 (100%)
Leptomeningeal disease	18 (52.9%)	3 (37.5%)
Treatment	Radiation—34 (100%) Chemotherapy—32 (94.1%)	Radiation—8 (100%) Chemotherapy—8 (100%)

^*^Includes basal ganglia and pineal gland.

LMD: leptomeningeal disease.

**Figure 3.**
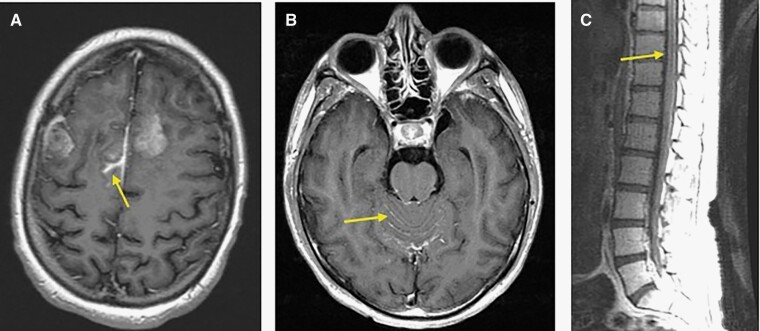
Imaging signs of LMD. (A) MRI from patient in the H3 G34 group (patient 19 in Table 2) showing peritumoral sulcal enhancement in the right parasagittal frontal lobe. (B) and (C) MRI of the brain (B) and spine (C) from a patient from the H3 K27 group (corresponding to patient 1 in Table 2) showing diffuse leptomeningeal enhancement in the cerebellar folia and brainstem surface as well as along the lower spinal cord and cauda equina.

The detailed characteristics of the 21 patients who developed LMD are described in Table 2. The median time from tumor diagnosis to LMD diagnosis was 12.9 months in the H3 K27 group and 5.6 months in the H3 G34 group. Only a minority of patients (4/19 patients with available data) had symptoms attributable to LMD at the time of this diagnosis. Most patients in the H3 K27 group had diffuse LMD (involving both the brain and spine) and were diagnosed through neuroimaging alone, and 2 patients (out of 8 tested) had a positive CSF cytology in addition to radiologic signs of LMD. LMD was localized in the 3 H3 G34 patients (peritumoral in 1 case, and distant to the original area of tumor in the other 2; Figure 3), and none of these patients had a lumbar puncture performed. Lumbar puncture for CSF analysis, including NGS, was performed in 8/18 LMD patients in the H3 K27 group, and an H3 mutation was detected in all cases.

**Table 2.** Clinical and pathological characteristics of patients with leptomeningeal disease.

		Sex	Age at TD	Months from TD to LMD diagnosis	Tumor location	LMD location	LMD symptoms	LMD diagnosis	CSF cytology	CSF H3 mutation on NGS (VAF)
H3 K27	1	M	21	0	Thalamus	Diffuse	No	MRIb + MRIs + CSF	Positive	Positive (0.54)
	2	F	20	4.4	Thalamus	Diffuse	No	MRIb + MRIs + CSF	Positive	Positive (0.25)
	3	M	22	14	Thalamus	Diffuse	Unknown^a^	MRIb + MRIs	Negative	Positive (0.09)
	4	M	12	6.5	Thalamus	Diffuse	Yes (back/thigh pain)	MRIb + MRIs	NP	NP
	5	M	9	8.2	Thalamus	Diffuse	Yes (back pain)	MRIb + MRIs	NP	NP
	6	F	45	13	Thalamus	Peritumoral	No	MRIb	NP	NP
	7	M	17	16.4	Thalamus	Diffuse	No	MRIb + MRIs	Negative	Positive (0.05)
	8	M	46	38.2	Thalamus	Distant	No	MRIb	NP	NP
	9	F	20	40.2	Thalamus	Peritumoral	No	MRIb	NP	NP
	10	M	31	12.8	Thalamus	Diffuse	Yes (urinary incontinence)	MRIb + MRIs	NP	NP
	11	M	44	1.9	Brainstem	Diffuse	No	MRIb + MRIs	Negative	Positive (0.26)
	12	F	6	5.8	Brainstem	Diffuse	Yes (back pain, constipation)	MRIb + MRIs	NP	NP
	13	M	9	28.3	Brainstem	Peritumoral	No	MRIb	NP	NP
	14	M	4	3.8	Brainstem	Diffuse	No	MRIb + MRIs	NP	NP
	15	M	27	23.6	Spine	Diffuse	No	MRIb + MRIs	Negative	Positive (0.32)
	16	M	31	44.4	Spine	Diffuse	No	MRIb + MRIs	Negative	Positive (0.85)
	17	F	33	7.4	Spine	Diffuse	No	MRIb + MRIs	Negative	Positive (0.80)
	18	F	18	39.4	Pineal gland	Peritumoral	Unknown^a^	MRIb	NP	NP
H3 G34	19	F	17	2.3	Frontal	Peritumoral	No	MRIb	NP	NP
	20	M	13	6.2	Frontal	Distant	No	MRIb	NP	NP
	21	M	32	5.6	Parietal	Distant	No	MRIs	NP	NP

^a^Patient was diagnosed with leptomeningeal disease outside of our institutions, and no details about symptoms at the time of diagnosis are available.

CSF: cerebrospinal fluid, F: female, LMD: leptomeningeal disease, M: male, MRIb: magnetic resonance imaging of the brain, MRIs: magnetic resonance imaging of the spine, NGS: next-generation sequencing, NP: not performed, TD: tumor diagnosis VAF: variant allelic frequency.

**Figure 4.**
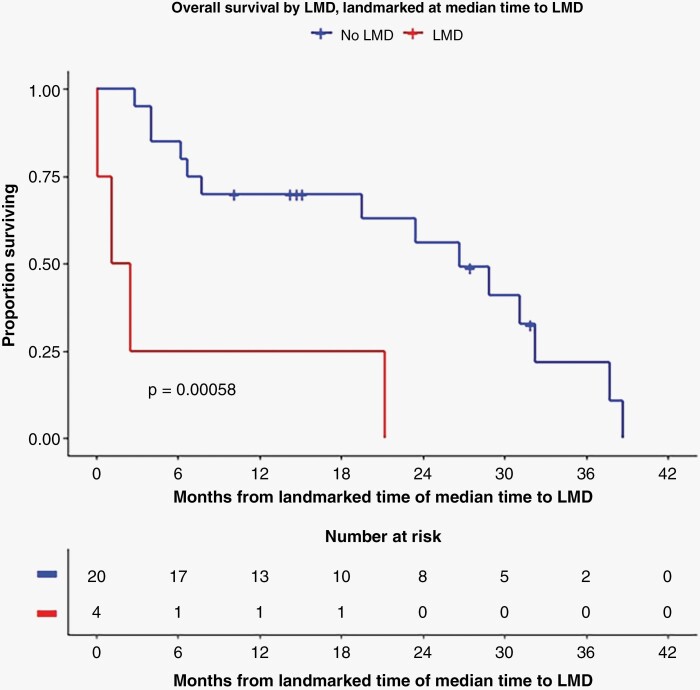
Overall survival of H3 K27 group according to LMD status (LMD in orange, no LMD in blue), landmarked to median time of LMD diagnosis.

Median overall survival for the H3 K27 group was 1.72 years (95% CI: 1.31–3.29) and 1.26 years (95% CI: 0.65–1.46) in the H3 G34 group; there was no significant difference in unadjusted survival according to development of LMD in the H3 KG34 group. However, the modeled risk of death was increased in the H3 K27 group for patients who developed LMD compared to those who did not develop LMD (hazard ratio (HR) for risk of death: 7.37, 95% CI: 2.98–18.23, *P* < .0001; Figure 4, landmarked Log-Rank *P*-value = .00058). In the H3 G34 group, there was no difference in the risk of death for patients who developed LMD (HR: 0.55, 95% CI: 0.10–3.09, *P* = .49). All patients received treatment with at least radiation therapy at initial diagnosis, and most patients also received chemotherapy (Table 1).

## Discussion

Our results show that the high risk of LMD previously reported in DIPG^5,6^ also extends to nonDIPG H3 K27-altered DMG, with half of the patients developing LMD throughout the disease course, including 15 asymptomatic patients among both groups. Given the small number of patients with H3 G34-mutant DHG in our cohort, the results observed in this group should be interpreted with caution; however, our data suggest an increased risk of LMD in these patients in comparison to a large retrospective study from our institution which demonstrated the incidence of LMD in adult patients with gliomas is around 5%.^11^

Our findings further support the notion that the presence of H3 mutations may predict a greater tendency to disseminate through the CSF compared to other gliomas, through unknown mechanisms. In the case of DMG, their midline location—and subsequent proximity to CSF—may be construed as contributing to their propensity to seed the leptomeningeal space, as opposed to tumor-specific molecular mechanisms. To address this question, we reviewed the incidence of LMD in all cases of brainstem glioma collected in the initial chart review for the aforementioned retrospective study (but not included in subsequent analysis for that study, as it excluded tumors in brainstem and spinal cord).^11^ Out of 102 patients with brainstem gliomas in this database, only 5 (4.9%) developed LMD during their disease, consistent with the incidence reported in gliomas in other locations and much smaller than the risk seen in histone-mutant gliomas in the present study. Of note, these 102 patients may include some H3 K27-altered DMG, as this database contained patients diagnosed between 2001 and 2016, when histone mutations were not comprehensively investigated or reported. Regardless, these data suggest that midline location may not be the only factor explaining the high frequency of LMD in H3 K27-altered DMG patients.

Detection of cell-free tumor-derived DNA (ctDNA) in CSF as a form of liquid biopsy may be of particular interest in the case of H3 K27-altered DMG, given the potential risks of surgically accessing midline brain structures and the presence of a disease-defining genetic mutation.^12^ In several small case series of H3 K27-altered DMG, *H3F3A* or *HIST1H3B* mutations were detected in the CSF in most patients (for whom the presence of LMD had not been specified), likely reflecting shedding of tumor DNA into the subarachnoid space due to their close proximity to this anatomical location.^13–17^ In our study, ctDNA and in particular histone mutations were detected in all patients with LMD that had undergone NGS (8/8), but, unfortunately, we do not have CSF data on patients without LMD to determine whether the rate of detection is significantly different between these 2 groups. It is unclear whether ctDNA in CSF could be used as a marker of true leptomeningeal dissemination, as opposed to simple shedding of unviable tumor material into the CSF. Our results suggest that VAF may not be a relevant marker in this scenario, as this parameter was substantially variable within our patients with LMD (range 0.05–0.85); nonetheless, more studies are needed to elucidate how tumor DNA could be best used in this population.

Our results also demonstrate that patients with H3 K27-altered DMG who develop LMD have a substantially reduced survival, with an increase in the risk of death by a factor of over 7 after developing LMD compared to nonLMD patients in the same group. This influence on survival in H3 K27-altered DMG argues in favor of attempting to diagnose LMD early, which will frequently require a high level of suspicion, as illustrated by the fact that only a minority of our patients with LMD had symptoms that could be attributed to this disease. Although we do not have data to determine whether an early diagnosis of LMD changes the trajectory of the disease, our data suggest that early consideration of LMD and ordering ctDNA CSF analysis and imaging of neuroaxis could be warranted and may positively impact patients and providers, at least by providing a more accurate context for prognostic discussions. We were unable to prove similar findings in the H3 G34-mutant DHG group, although it is unclear whether this lack of association is due to the small sample size.

A salient finding of our study is the description of an uncommon, noncanonical mutation in DMG. One of our patients had a mutation in gene *H3F3B*, which encodes a histone protein identical to that encoded by *H3F3A* and is frequently mutated in chondroblastoma.^18^ In our patient, the mutation affected codon 27 and resulted in a lysine-to-isoleucine substitution (K27I); this substitution has been described as an uncommon variant in DMG, affecting *H3F3A* and resulting in the same loss of trimethylation that mediates the pathogenic effect of the more common K27M mutation.^19^ A single case of this same mutation in the gene *H3F3B* has been reported in a spinal cord glioma,^20^ and our description of this mutation in a patient with a clinical picture entirely consistent with DMG further supports the notion that this represents a non-canonical pathogenic mutation for this tumor type. In addition, one of our patients had both an H3 K27 and an H3 G34 mutation, and as explained above was analyzed in the K27 group given phenotype (thalamic tumor) was more consistent with DMG than with DHG. To our knowledge, this coexistence of mutations has not been previously reported, and its significance is unclear.

One of the limitations of the present study is its retrospective nature. In addition, only patients who had had a tissue diagnosis that was analyzed with the NGS assay were included in our study, which may limit the generalizability of our findings. As a consequence, our series includes only a small subset of 4 DIPG patients, since biopsies for these patients are not required for diagnosis, and outside of clinical trials they are generally reserved for patients with atypical age, clinical or imaging findings; on the other hand, this enrichment for non-DIPG patients allows us to better characterize LMD in this subset of DMG patients, for whom LMD incidence and characteristics are not well established in the literature. Among the strengths of our study, we highlight the relatively large number of patients with histone-mutant tumors, the availability of detailed genomic information from both tumor and CSF confirming *H3* mutation in all cases, *TP53* in a subset of cases, and co-existence of *PDGFRA, FGFR1, and BRAF* in some patients, underscoring the importance of comprehensive NGS as opposed to isolated *H3* testing.

In summary, our data suggest that patients with histone-mutant gliomas, including H3 K27-altered DMG and H3 G34-mutant DHG, are at an increased risk of LMD compared to other glioma patients. In addition, patients with H3 K27-altered DMG that develop LMD may be at risk of worsened survival. Given this heightened risk, clinicians should consider a lower threshold to perform whole neuroaxis imaging as well as CSF analysis in patients with histone-mutant gliomas. If CSF analysis is pursued, NGS analysis can be a valuable tool to provide updated information on the tumor mutational profile at the time of LMD diagnosis, even for cases with negative CSF cytology. In order to establish definitive clinical utility of CSF profiling in these patients, additional follow-up prospective studies that are sufficiently powered should be conducted. Further research will shed light on the broader implications and impact that these additional measures may have in this population of patients through prospective studies.

## Supplementary Material

vdad068_suppl_Supplementary_Table

## Data Availability

The Next-Generation Sequencing results have been made publicly available through cBioportal http://www.cbioportal.org/. The rest of the materials and data generated in this manuscript will be shared upon reasonable request.
