# Supplementary material for: Leptomeningeal disease in histone-mutant gliomas
Source: Neurooncol Adv. 2023 May 29;5(1):vdad068. doi: 10.1093/noajnl/vdad068 (PMC10281361; doi:10.1093/noajnl/vdad068)
Supplement: vdad068_suppl_Supplementary_Table [file vdad068_suppl_supplementary_table.docx]

|  | | | **Sex** | **Age at TD** | **Tumor location** | **Type of RT** | **Chemotherapy agents** |
| --- | --- | --- | --- | --- | --- | --- | --- |
| **H3 K27** | **LMD** | 1 | M | 21 | Thalamus | CSI | TMZ, CT, BEV, CCNU |
|  |  | 2 | F | 20 | Thalamus | IMRT | TMZ, BEV, BCNU |
|  |  | 3 | M | 22 | Thalamus | IMRT | TMZ, BEV |
|  |  | 4 | M | 12 | Thalamus | IMRT | CT |
|  |  | 5 | M | 9 | Thalamus | IMRT | TMZ |
|  |  | 6 | F | 45 | Thalamus | IMRT | TMZ, BEV, irinotecan |
|  |  | 7 | M | 17 | Thalamus | Focal Proton | TMZ, BEV |
|  |  | 8 | M | 46 | Thalamus | IMRT | TMZ, abemaciclib, pembrolizumab |
|  |  | 9 | F | 20 | Thalamus | IMRT | TMZ, BEV, CT, everolimus |
|  |  | 10 | M | 31 | Thalamus | IMRT | TMZ, BEV |
|  |  | 11 | M | 44 | Brainstem | IMRT | TMZ, BEV |
|  |  | 12 | F | 6 | Brainstem | IMRT | None |
|  |  | 13 | M | 9 | Brainstem | Focal Proton | CT |
|  |  | 14 | M | 4 | Brainstem | IMRT | CT |
|  |  | 15 | M | 27 | Spine | IMRT | TMZ, CCNU, BEV, pembrolizumab |
|  |  | 16 | M | 31 | Spine | IMRT | TMZ, CT, BEV, trametinib |
|  |  | 17 | F | 33 | Spine | IMRT | TMZ, CCNU, carboplatin, BEV, pembrolizumab |
|  |  | 18 | F | 18 | Pineal gland | IMRT | TMZ, BEV, irinotecan, panabinostat |
|  | **No LMD** | 19 | M | 38 | Thalamus | IMRT | TMZ |
|  |  | 20 | M | 53 | Thalamus | Unknown | TMZ |
|  |  | 21 | M | 70 | Thalamus | IMRT | TMZ |
|  |  | 22 | F | 14 | Thalamus | IMRT | CT |
|  |  | 23 | F | 12 | Thalamus | IMRT | CT, dabrafenib/trametinib |
|  |  | 24 | M | 21 | Thalamus | IMRT | TMZ, CT, BEV |
|  |  | 25 | M | 24 | Thalamus | IMRT | TMZ, CT, BEV |
|  |  | 26 | F | 7 | Brainstem | IMRT | TMZ +PARPi, etoposide, temsirolimus, dasatanib, thalidomide |
|  |  | 27 | F | 10 | Brainstem | IMRT | CT, BEV |
|  |  | 28 | F | 21 | Brainstem | IMRT | Ipilimab/nivolumab, CT |
|  |  | 29 | F | 10 | Brainstem | IMRT | CT, ipilimab/nivolumab |
|  |  | 30 | M | 8 | Brainstem | IMRT | CT |
|  |  | 31 | M | 21 | Brainstem | IMRT | Dabrafenib/trametinib, BEV, CT |
|  |  | 32 | F | 64 | Spine | IMRT | TMZ, BEV |
|  |  | 33 | F | 42 | Spine | Focal Proton | TMZ |
|  |  | 34 | M | 53 | Basal ganglia | IMRT | TMZ |
| **H3 G34** | **LMD** | 35 | F | 17 | Frontal | IMRT | TMZ |
|  |  | 36 | M | 13 | Frontal | IMRT | TMZ, BEV, CCNU, dasatinib |
|  |  | 37 | M | 32 | Parietal | IMRT | CT, TMZ, BEV, pembrolizumab, carboplatin |
|  | **No LMD** | 38 | M | 24 | Parietal | IMRT | TMZ |
|  |  | 39 | M | 19 | Parietal | IMRT | TMZ, BEV, everolimus |
|  |  | 40 | M | 23 | Temporal | IMRT | TMZ |
|  |  | 41 | M | 9 | Temporal | IMRT | TMZ, BEV, CCNU |
|  |  | 42 | M | 26 | Occipital | IMRT | TMZ, CT, BEV, CCNU |

**Supplementary Table 1**: Treatment information for all patients. BCNU: carmustine, BEV: bevacizumab, CCNU: lomustine, CSI: craniospinal irradiation, CT: clinical trial, F: female, IMRT: intensity-modulated radiation therapy, LMD: leptomeningeal disease, M; male, PARPi: poly(ADP-ribose) polymerase inhibitor, RT: radiation therapy, TD: tumor diagnosis, TMZ: temozolomide.
